# CD90 determined two subpopulations of glioma-associated mesenchymal stem cells with different roles in tumour progression

**DOI:** 10.1038/s41419-018-1140-6

**Published:** 2018-10-27

**Authors:** Qing Zhang, Dong-Ye Yi, Bing-Zhou Xue, Wan-Wan Wen, Yin-Ping Lu, Ahmed Abdelmaksou, Min-xuan Sun, De-tian Yuan, Hong-Yang Zhao, Nan-Xiang Xiong, Wei Xiang, Peng Fu

**Affiliations:** 1 Department of Neurosurgery,Union Hospital, Tongji Medical College, Huazhong University of Science and Technology, Wuhan, 430022 China; 2 Department of Cardiology, Beijing Anzhen Hospital, Capital Medical University, No. 2, Anzhen Road, Chaoyang District, Beijing, 100029 China; 3 Institute of Infection and Immunology, Union Hospital, Tongji Medical College, Huazhong University of Science and Technology, Wuhan, 430022 China; 4 Department of Neurosurgery, Faculty of Medicine, Helwan University, Cairo, 11435 Egypt; 5 Jiangsu Key Lab of Medical Optics, Suzhou Institute of Biomedical Engineering and Technology, Chinese Academy of Sciences, Suzhou, 215163 China

## Abstract

Human glioma-associated mesenchymal stem cells (gbMSCs) are the stromal cell components that contribute to the tumourigenesis of malignant gliomas. Recent studies have shown that gbMSCs consist of two distinct subpopulations (CD90^+^ and CD90^−^ gbMSCs). However, the different roles in glioma progression have not been expounded. In this study, we found that the different roles of gbMSCs in glioma progression were associated with CD90 expression. CD90^high^ gbMSCs significantly drove glioma progression mainly by increasing proliferation, migration and adhesion, where as CD90^low^ gbMSCs contributed to glioma progression chiefly through the transition to pericytes and stimulation of vascular formation via vascular endothelial cells. Furthermore, discrepancies in long non-coding RNAs and mRNAs expression were verified in these two gbMSC subpopulations, and the potential underlying molecular mechanism was discussed. Our data confirm for the first time that CD90^high^ and CD90^low^ gbMSCs play different roles in human glioma progression. These results provide new insights into the possible future use of strategies targeting gbMSC subpopulations in glioma patients.

## Introduction

Gliomas are the primary central nervous system tumours with the highest incidence despite progress made in combination treatment using surgical, radiotherapy and chemotherapy approaches^1,2^. Better understanding of the tumour microenvironment will enable pursuit and development of a promising therapeutic strategy for gliomas^3,4^.

Generally, the tumour microenvironment consists of tumour cells, fibroblasts, endothelial cells, mesenchymal stem cell (MSCs), and inflammatory cells as well as cytokines and chemokines secreted by tumour and stromal cells^3^. In gliomas, MSCs can be recruited by some factors into the tumour microenvironment and modulate tumour development^5^. Our team reported that glioma-associated MSCs (gbMSCs) had classical MSC surface markers (CD105, CD73, CD90 and CD44) but lacked expression of CD14, CD34 and CD45. Gb-MSCs show plastic adherent morphology and have the capacity to differentiate into osteoblasts, adipocytes and chondroblasts in vitro^6,7^. The percentage of gbMSCs in high-grade glioma samples is closely related to their survival within GBM patients^8^. Furthermore, we found that human gbMSCs were integral components in the pericyte transition and tumour vascular formation.^6^ Some reports have demonstrated that gbMSCs can increase glioma stem cell self-renewal and proliferation via secretion of exosomes and factors.^9^

Recent reports found that gbMSCs could be divided into two subtypes according to CD90 expression (CD90^**+**^ gbMSCs and CD90^**−**^ gbMSCs). CD90^**−**^ cells are regarded as more active in glioma vascularization and immunosuppression than their CD90^**+**^ counterparts, and CD90^**−**^ and CD90^**+**^ gbMSCs differ greatly in their mRNA expression patterns^10^. However, the biological properties of these two distinct subpopulations and their effects on glioma have not been fully elaborated.

In this study, we elaborately sorted two distinct MSC-like cell populations from gbMSCs according to differences in CD90 surface marker expression and investigated the different roles of these two gbMSC subpopulations in glioma progression.

## Materials and methods

### Tumour samples

Human brain tumour samples were obtained from the Neurosurgery Department at Union Hospital in Wuhan, China, after patients with glioma provided informed consent. The specimens were reviewed by a neuropathologist to assess the grade and tumour type before the assays were performed (Table1). Typically, cell separation was performed within 1 h of tumour resection.

**Table1:** Characteristics of 14 patients with gliomas used for gbMSC isolation in the current study

Specimen	Age (years)^a^	Sex	Pathological diagnosis	Grade	Passage at experiment	CD90 expression in gbMSCs (%)
AC-11	29	M	Astrocytoma	Ill	3	20.1
GBM-13	53	F	GBM	IV	2	21.3
AC-16	58	F	Astrocytoma	Ill	2	19.2
ODG-18	36	M	Oligodendroglioma	II	3	13.5
GBM-21	35	F	GBM	IV	2	21.7
GBM-23	59	M	GBM	IV	3	25.9
AC-24	45	M	Astrocytoma	Ill	3	18.9
GBM-31	58	M	GBM	IV	3	19.8
GBM-32	56	M	GBM	IV	4	23.8
AC-35	62	F	Astrocytoma	II	2	17.9
GBM-35	79	F	GBM	IV	2	22.1
GBM-71	61	F	GBM	IV	4	20.3
AC-73	36	F	Astrocytoma	Ill	3	19.8
AV-76	38	M	Astrocytoma	IV	2	21.5

^a^At the time of diagnosis

### Isolation and culture of human gbMSCs

The sample was transferred to a Petri dish, washed three times in phosphate-buffered saline (PBS, HyClone, USA), and cut into 1-mm^3^ pieces. Next, 0.25% trypsin (BIYUNTIAN, China) was added to the tumour specimens. Then, the single pieces were digested for 20 min, filtered with a 70-µm nylon mesh (Corning, USA) and centrifuged at 1000 rpm for 15 min. The mononuclear cells were collected by Ficoll (2:1 Genview, USA) density gradient centrifugation at 1500 rpm for 20 min without braking. Single cells were re-suspended in DMEM (HyClone, USA) containing 10% foetal bovine serum (BI, Israel) and 100 U/ml of penicillin/streptomycin (GibcoBRL, Grand Island, NY, USA), seeded into a 25-cm^2^ culture flask (Corning, USA) and incubated at 37 °C and humidity with 5% CO_2_. The medium was changed 1–3 times every week. Cells at 70–80% confluency were passaged using Accutase (StemCell, Canada) and used for experiments at passages 2 to 3.

### Cell lines

U87-MGs were purchased from the American Type Culture Collection (ATCC, Gaithersburg, MD, USA), and human umbilical vein endothelial cells (HUVECs) were purchased from Lonza (MD, USA). The U87-MGs and HUVECs were cultured in Dulbecco’s modified Eagle’s medium (DMEM) (HyClone, USA) and RPMI 1640 medium (Gibco, USA), respectively, supplemented with 10% foetal bovine serum (FBS; BI, Israel) and 100 U/ml of penicillin/streptomycin (GibcoBRL, Grand Island, NY, USA) in humidified atmosphere at 37 °C with 5% CO_2_.

### Conditioned media

For all experiments, U87-MGs, CD90^high^ gbMSCs and CD90^low^ gbMSCs were seeded into T25 tissue culture flasks in DMEM with 10%FBS containing penicillin (100 U/mL)/streptomycin (100 mg/mL) (GibcoBRL, Grand Island, NY, USA). When the cell density reached 50–60% confluency, the medium of the U87-MGs was replaced with serum-free medium (DMEM) and DMEM supplemented with 10% FBS, the medium of the CD90^high^ and CD90^low^ gbMSCs was replaced with serum-free medium (DMEM), and the cells were cultured for 72 h. Conditioned media were collected from the flasks and centrifuged at 1000 × *g* for 10 min to remove cells and cellular debris. Afterward, the collected conditioned media (CD90^high^ CM, CD90^low^ CM, 0%gb-CM, and S-gb-CM) were stored at −20 °C prior to use.

### Differentiation of gbMSCs

The gbMSCs were adipogenically, osteogenically and chondrogenically induced using ready-to-use differentiation media (all from Stemcell, Canada) following the manufacturer’s instructions. Adipogenic differentiation was evaluated by oil red O staining, osteogenic differentiation was evaluated by Alizarin red staining and chondrogenic differentiation was evaluated by Alcian blue staining (all from Sigma, USA). The specific steps were as follows.

For osteoblast differentiation, the cells were cultured in growth medium in a six-well plate and incubated at 37 °C with 5% CO_2_ until they were approximately 70–80% confluent. Next, the medium was replaced by complete osteogenic stimulatory medium, the cells were incubated at 37 °C and the medium was changed every 3 days. The differentiation assay took approximately 3 weeks. Osteogenic differentiation was visualized by Alizarin red S staining.

For adipocyte differentiation, the cells were cultured in standard medium in a six-well plate at 37 °C and 5% CO_2_ until they were approximately 90–100% confluent. Then, the medium was aspirated and replaced with complete adipogenic differentiation medium, which was changed every 3 days. The differentiation assay took approximately 14 days. Adipogenic differentiation was visualized by oil red O staining.

For chondrocyte differentiation, cell pellets were grown in chondrogenesis induction medium for 21 days, and half of the medium was changed during differentiation. Histological sections of the pellet were generated by fixing the pellets in 10% formalin for 30 min at room temperature (15–25 °C), followed by subsequent standard paraffin embedding methods and staining of 6-µm-thick sections with Alcian blue.

### Magnetic activated cell sorting (MACS) of the gbMSCs

gbMSCs grown in good condition were used for the MACS experiment. First, the cells were immuno-labelled with CD90 microbeads (Miltenyi, Germany). Magnetic labelling was performed strictly according to the manufacturer’s instructions. Briefly, the gbMSCs were digested using Accutase (Stemcell, Canada) and centrifuged at 1500 rpm for 6 min.The cell pellet was re-suspended in 80 µL of precooled sorting buffer per 10^7^ total cells, and 20 µL of CD90 MicroBeads was added per 10^7^ total cells. Then, the solution was mixed well and incubated for 15 min in the dark in the refrigerator (2−8 °C). The cells were washed by adding 2 mL of buffer per 10^7^ cells and centrifuged at 1500 rpm for 6 min. The supernatant was aspirated completely. The cells were re-suspended in 500 μL of sorting buffer. Magnetic separation was performed using the autoMACS Pro Separator (Miltenyi, Germany).Ultimately, we obtained CD90^high^ gbMSCs and CD90^low^ gbMSCs.

### Flow cytometry

Flow cytometry analysis was performed using fluorochrome-conjugated antibodies. Briefly, different passages of gbMSCs, CD90^high^ gbMSCs and CD90^low^ gbMSCs were trypsinized and washed in PBS, and then the pellets were re-suspended in fluorescent-activated cell sorting (FACS) buffer. These single-cell suspensions were incubated in the dark at 4 °C for 30 min with PE-, FITC-, PE-Cy7-, APC-Cy7-,PerCP- and APC-conjugated antibodies against human CD73, CD105, CD90, CD44, CD13, CD34, CD31, Desmin, α-SMA (from eBioscience, USA), NG2 and PDGFR-β (from R&D Systems, USA). Then, the cells were centrifuged, re-suspended in PBS and analysed using a flow cytometer (BD Biosciences). The data were collected and analysed using the FlowJo (TreeStar, Ashland, OR, USA) software.

### Cell proliferation assay

Cell viability was analysed using the Cell Counting kit-8 (CCK-8 Kit, Dojindo Laboratories, Japan). U87 cells (3000/well) was seeded into a 96-well plate and cultured overnight. Then, the medium was replaced with 100 µl of serum-free medium (0%DMEM), serum-free CD90^high^ gbMSC-conditioned medium (CD90^high^ CM), or serum-free CD90^low^ gbMSC-conditioned medium (CD90^low^ CM)and cultured for 1, 2, 3, or 4 days.CD90^low^and CD90^high^ gbMSCs were seeded into 100 µl of serum-free medium supplemented with 10% FBS (10%DMEM) and cultured for 1, 2, 3, or 4 h. At various time points, 10 µl of CCK-8 was added to each well and incubated for 2 h at 37 °C with 5% CO_2_. Then, the absorbance of each well was measured at 450 nm using a microplate reader (PerkinElmer, USA). At least three wells were used for each sample in different media. The assays were repeated at least three times.

### Adhesion assay

U87 cells were treated with CD90^high^ CM and CD90^low^ CM for 48 h. Then, the cells were seeded at a density of 1 × 10^4^ cells/well in 96-well plates pre-coated with matrix adhesive and incubated with 0%DMEM, CD90^high^ CM and CD90^low^ CM for 1 h at 37 °C in an atmosphere enriched with 5% CO_2_. The non-adherent cells were removed by washing carefully three times with PBS, and the cultures were incubated in 100 µl of medium with 10 µl of CCK8 (Dojindo Laboratories, Japan) for 2 h. Cell adhesion was analysed by measuring the optical density (OD) at 450 nm in a microplate reader (PerkinElmer, USA). At least three wells were used for each sample in different media. The assays were repeated at least three times.

### Migration assays

#### Transwell chamber assay

The migration capacity of the U87 cells was evaluated in 24-well plates with Transwell inserts with an 8-µm pore size (BD FALCON, USA). U87 cells (5 × 10^4^/ml) in 100 µl of serum-free DMEM were added to the upper chamber, and 800 µl of the tested samples (0%DMEM, CD90^high^ CM and CD90^low^ CM) was placed in the lower chambers. Cell migration was allowed for 24 h at 37 °C with 5% CO_2_. Following incubation, the media were aspirated, and the cells remaining on the upper surface of the polycarbonate membrane were removed with a cotton swab. The cells that migrated to the lower surface were stained with Giemsa for 20 min. Cell counting was performed under an inverted microscope by two researchers independently. The average numbers of migrated cells were determined by counting the cells in 5 random high-power fields (x200).

#### Wound-healing assay

A wound-healing assay was used to evaluate the migration ability of the CD90^high^and CD90^low^gbMSCs in different media in vitro. The cells were incubated in 6-well plates until they reached 90–100% confluence. Then, cross lines were carefully made using a 10-µl pipette tip, and the debris was washed away with PBS. The medium was replaced with serum-free medium (0%DMEM), standard medium (10%DMEM), serum-free glioblastoma-conditioned medium (0%gb-CM) and standard glioblastoma-conditioned medium (S-gb-CM). The areas of the scratch wounds were photographed with an Olympus microscope at 0 and 8 h. The assays were performed in triplicate at least three times, and the data were analysed using the Image J software (NIH, USA).

### Immunochemistry and Immunofluorescence

For immunochemistry, tumour tissue specimens were fixed in 4% paraformaldehyde and embedded in paraffin after collection from sacrificed mice. The tissue sections were cut and dewaxed, and then antigens were retrieval. The slides were rinsed in PBS, incubated overnight at 4 °C with diluted anti-CD31 (1:50) and anti-Ki67 (1:800) antibodies (Proteintech, WuHan, China), and then incubated with an HRP-conjugated secondary antibody (1:1, Boster, WuHan, China). Binding was detected using a DAB solution (Boster, WuHan, China). The tissues were counterstained using haematoxylin (Boster, WuHan, China). Images of the stained tissue samples were obtained using an Olympus microscope.

For immunofluorescence, human GBM surgical specimens were harvested and fixed with 4% paraformaldehyde, and sections were prepared for immunostaining. Nonspecific staining was blocked by pre-incubation of these sections in goat serum diluted with PBS for 30 min at room temperature. The primary antibodies used were as follows: goat anti-CD105 polyclonal antibody (1:25, R&D Systems, USA) and rabbit anti-CD90 monoclonal antibody (1:25, Boster, WuHan, China). After incubation with the primary antibody overnight at 4 °C, the sections were rinsed several times with PBS and incubated with the appropriate secondary antibodies at room temperature for 1 h. The secondary antibodies used were as follows: Cy3-conjugated goat anti-rabbit and FITC-conjugated goat anti-goat antibodies (1:100, all from Boster, WuHan, China). After washing in PBS, the sections were counterstained with DAPI (Beyotime, WuHan, China) and mounted with anti-fade mounting medium. Immunofluorescence microscopy was performed with an Olympus microscope.

### Tube formation assay

Growth factor-reduced Matrigel (BD, USA) was added to a flat-bottomed, pre-cooled, 96-well plate. After incubation at 37 ℃ with 5% CO_2_ for 1 h. CD90^high^ and CD90^low^ gbMSCs were labelled using Calcein AM (Tocris, USA) and DiO (Yeasen, Shanghai, China). Calcein AM-labelled CD90^high^ and CD90^low^ gbMSCs were seeded (2 × 10^4^/well) into wells containing serum-free medium (0%DMEM), standard medium (10%DMEM), serum-free glioblastoma-conditioned medium (0%gb-CM) or standard glioblastoma-conditioned medium (S-gb-CM). HUVECs (2 × 10^4^/well) were seeded into wells containing 0%DMEM, CD90^high^ CM and CD90^low^ CM. The HUVECs (2 × 10^4^/well) were co-cultured with DiO-labelled CD90^high^ and CD90^low^ gbMSCs (1 × 10^4^/well) in wells containing serum-free glioblastoma-conditioned medium (0%gb-CM). Then, the 96-well plate was incubatedat 37 °C in 5% CO_2_. Three wells were used for each medium sample. After 6 h, tube formation was photo documented with an Olympus microscope. Capillary-like tube formation and the attachment of CD90^high^ and CD90^low^ gbMSCs onto the HUVECs were analysed in three random fields of view per well using the ImageJ software (NIH, USA).

### ELISA assay

The VEGF, bFGF, SDF-1α, CCL5, MMP9 and IL-6 levels in the supernatants of each medium sample (0%DMEM, CD90^high^ CM and CD90^low^ CM) were measured using their respective ELISA kits (Neobioscience, China). All procedures were performed as described in the manufacturer’s instructions. The absorbance was measured at 450 nm.Three wells were used for each medium sample.The assays were repeated three times.

### RNA extraction and Clariom D microarray

CD90^high^ and CD90^low^ gbMSCs were cultured in standard medium until approximately 90% confluent. Total RNA was extracted from three different batches of CD90^high^ gbMSCs and three corresponding CD90^low^ gbMSCs cultured in standard medium using the TRIzol reagent (Invitrogen, USA). After the total RNA quality was analysed with a NanoDrop (Thomas Fisher Scientific), cDNA was prepared using the GeneChip WT PLUS Kit and hybridized onto Affymetrix GeneChip® Clariom D arrays (Affymetrix, Santa Clara, CA, USA), which were washed and scanned according to Affymetrix’s instructions (Fluidics Protocol FS450_0003). The microarray data were measured and summarized using the Clariom D QC tool software (Affymetrix, Santa Clara, CA, USA).

### In vivo experiment

Male BALB/c-nu mice (4–6 weeks old; Beijing Vital River Laboratory Animal) were kept in the animal facilities at Huazhong University of Science and Technology and maintained under specific pathogen-free conditions. Intraperitoneal (IP) injections of chloral hydrate (2.5 ml/kg) were used to anaesthetize the animals in all experiments. All animal procedures were conducted in accordance with institutional guidelines under approved protocols. For the intracranial implantation of syngeneic U87 glioma cells in the brains of the BALB/c-nu mice, the animals were stereotactically inoculated with a CD90^high^ CM or CD90^low^ CM suspension containing 5 × 10^5^ U87 cells into the right frontal lobe (2 mm lateral and 1 mm anterior to the bregmaat a 3.5-mm depth from the skull base) using a Hamilton syringe (Hamilton Company, USA). The mice were sacrificed 28 days after injection. The tumour volumes of the nude mice in each group were calculated as *V* = 1/2*LW*^2^(*L* = tumour length, *W* = tumour width).

Additionally, we took advantage of the full list of available datasets presented on the GlioVis homepage (http://gliovis.bioinfo.cnio.es/) to analyse the overall survival of patients with gliomas in groups with differing CD90 expression levels from TCGA GBM dataset.

### Statistical analysis

The statistical analyses were performed with GraphPad Prism. Unless specifically noted, all data were representative of at least three separate experiments. Error bars represent the standard deviations (SDs) and were calculated using Prism. The specific statistical tests used were a *t* test for single comparisons or ANOVA followed by Tukey’s test for multiple comparisons, and all *P* values < 0.05 were considered statistically significant. GraphPad Prism was used to compare two survival curves with the log-rank test.

## Results

### Characteristics of CD90^high^ and CD90^low^ gbMSCs

**Fig. 1:**
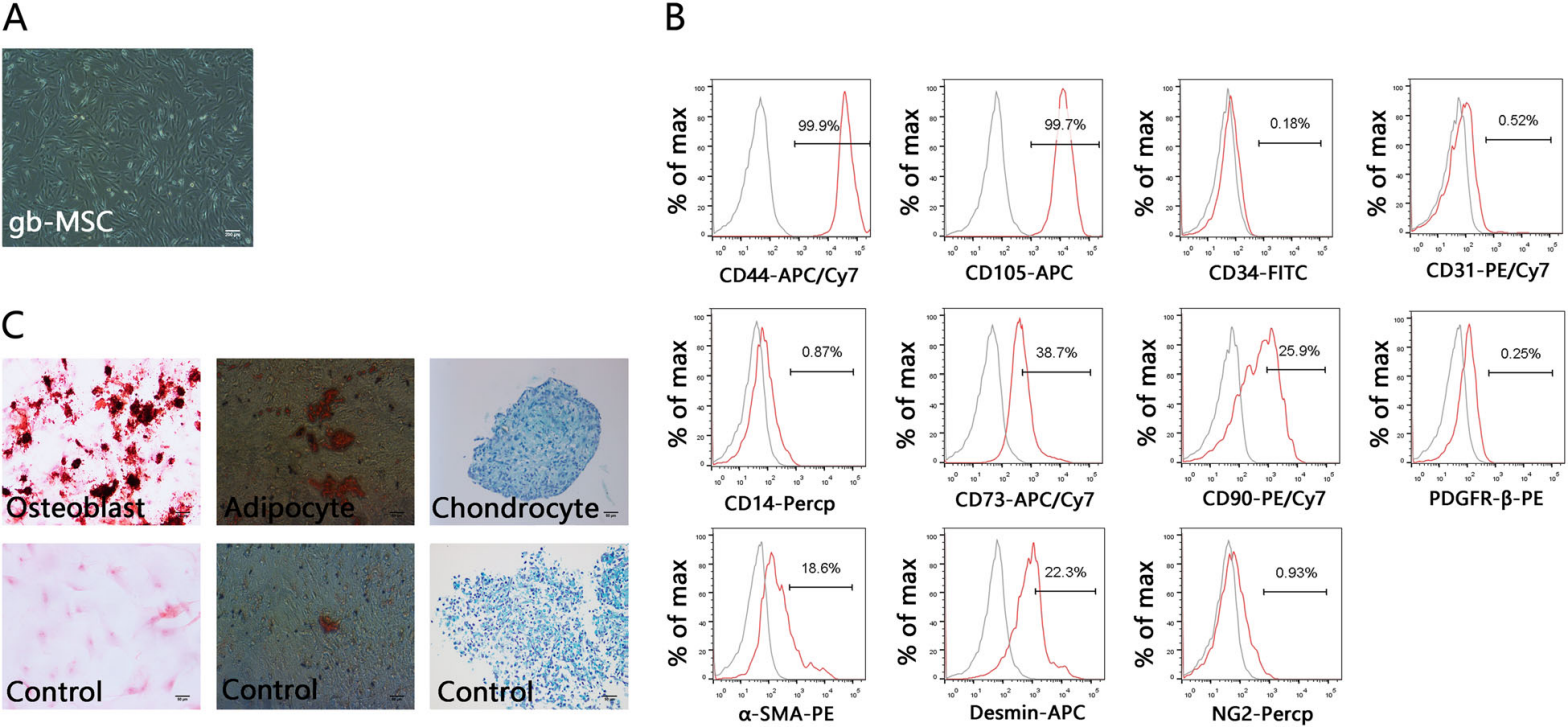
Isolation and characterization of gbMSCs derived from human high-grade glioma tissues. **a** Adherent growth pattern of gbMSCs cultured in 10% DMEM (×40, scale bars = 200 μm). **b** FACS analysis of typical gbMSCs (*n* ≥ 3) in culture. **c** Tri-lineage differentiation of gbMSCs: gbMSCs were treated with specific conditions for osteogenic differentiation (upper left), adipogenic differentiation (upper middle), and chondrogenic differentiation (upper right) (×200, scale bars = 50 μm). The lower panels show staining of cells grown in MSC medium as a control

gbMSCs were successfully isolated from fresh tumour tissues from patients with different grade gliomas, as shown in Table1. These gbMSCs from astrocytomas and glioblastomas showed similar classical MSC characteristics in vitro. They displayed a fibroblastic morphology and grew adherent to flasks in standard medium (Fig. 1a). The flowcytometric analysis showed that the gbMSCs expressed MSC markers, including CD73, CD105, CD44, and CD90, but not CD31, CD34, CD14, NG2 and PDGFβ-R; moreover, slight α-SMA and desmin expression was observed (Fig. 1b). The potential of gbMSCs to differentiate into adipocytes, osteoblasts and chondrocytes was tested in vitro using specific stimuli to promote adipogenesis, osteogenesis and chondrogenesis, respectively. Adipogenic differentiation was observed with oil red O staining, osteogenic differentiation was observed with Alizarin red staining and chondrogenic differentiation was observed with Alcian blue staining (Fig. 1c). The gbMSCs were able to differentiate into adipocytes, osteoblasts and chondrocytes.

**Fig. 2:**
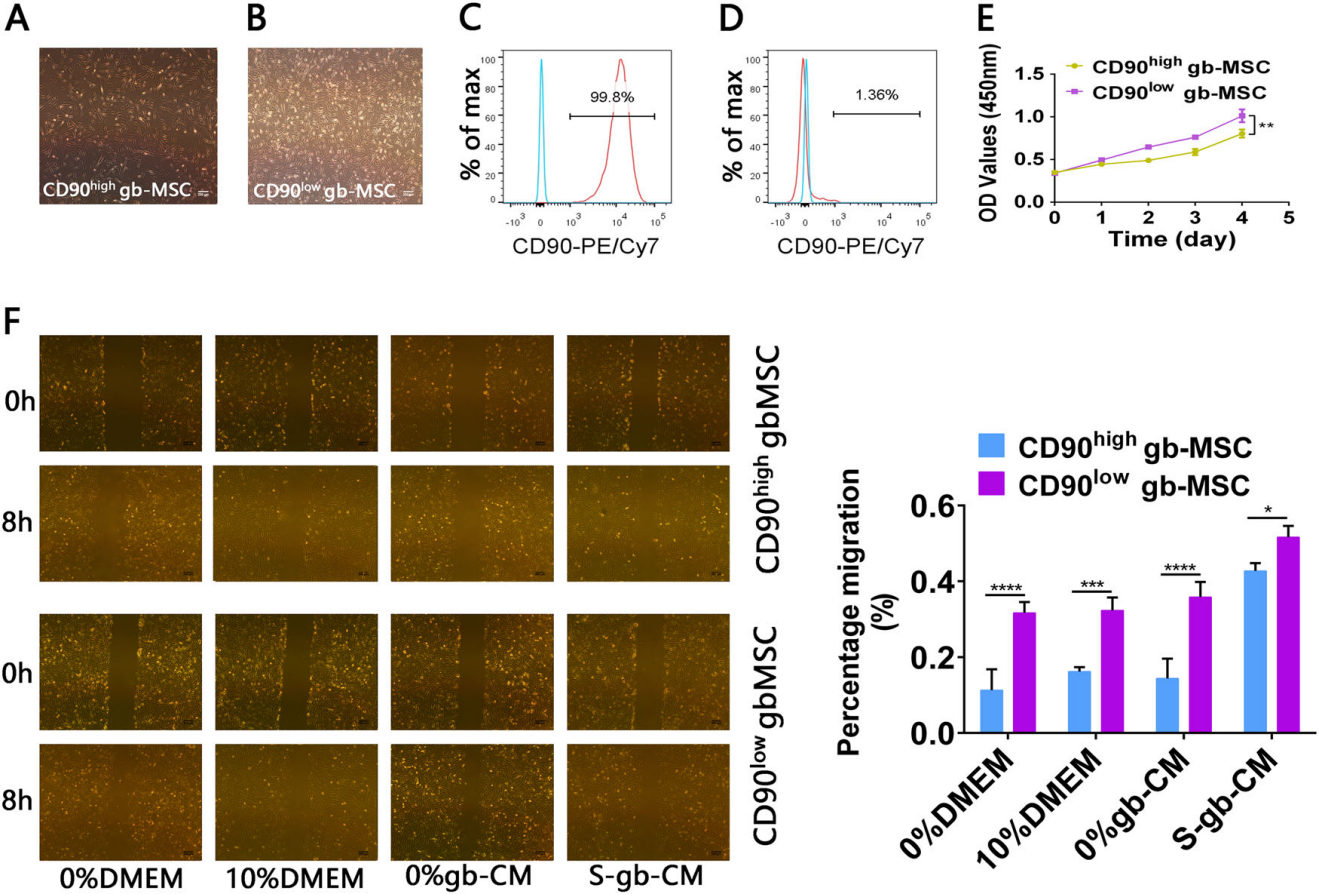
Characteristics of CD90^high^and CD90^low^ gbMSCs cultured in vitro. **a**, **b** Adherent growth patterns of CD90^high^ and CD90^low^ gbMSCs cultured in 10% DMEM (×40, scale bars = 200 µm). **c** FACS analysis of sorted CD90^high^ gbMSCs (*n* ≥ 3). **d** FACS analysis of sorted CD90^high^ gbMSCs (*n* ≥ 3). **e** Growth of CD90^high^ and CD90^low^ gbMSCs cultured in 10% DMEM (*n* ≥ 3). *P < 0.05, **P < 0.01. **f** Wound-healing assay of CD90^high^ and CD90^low^ gbMSCs (*n* ≥ 3) for 8 h with different media (×40, scale bars = 200 µm). *P < 0.05, **P < 0.01, ***P < 0.001 and ****P < 0.0001

Similarly, CD90^high^ and CD90^low^ gbMSCs all showed typical adherent growth in vitro (Fig. 2a, b). gbMSCs associated with CD90 expression were verified by flowcytometric analysis (Fig. 2c, d). Additionally, we found that their growth was significantly different and that CD90^low^ gbMSCs cultured in 10%DMEM grew faster than CD90^high^ gbMSCs in vitro (Fig. 2e). Furthermore, the migration capacity of the CD90^low^ gbMSCs incubated in different conditioned media was significantly stronger than that of CD90^high^ gbMSCs (Fig. 2f).

### CD90^high^ gbMSCs significantly promote glioma progression in vitro

**Fig. 3:**
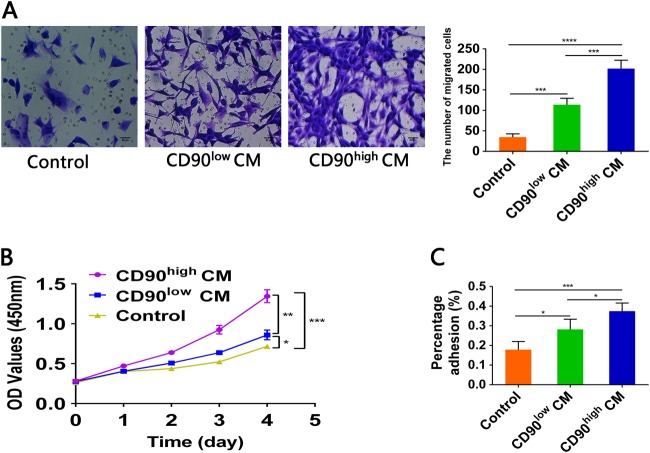
The migration, proliferation and adhesion capacities of U87 cells incubated with different media in vitro. **a** Transwell assay of U87 cells cultured for 24 h in different media (*n* ≥ 3) (serum-free medium, CD90^low^ CM and CD90^high^ CM). *P < 0.05, **P < 0.01, ***P < 0.001 and ****P < 0.0001. Serum-free medium was used as a control. **b** CCK8 assay of U87 cells (*n* ≥ 3) to evaluate proliferation in different media in vitro. U87 cells were incubated in 0%DMEM, CD90^high^ CM and CD90^low^ CM. *P < 0.05, **P < 0.01, ***P < 0.001. Serum-free medium was used as a control. **c** Adhesion assay to estimate the effect of 0%DMEM, CD90^high^ CM and CD90^low^ CM on U87 cell adhesion. (*n* ≥ 3) *P < 0.05, **P < 0.01, ***P < 0.001. Serum-free medium were used as a control

To demonstrate that CD90^high^ gbMSCs significantly promoted glioblastoma growth, migration and adhesion in vitro, experiments were performed using CCK-8, Transwell chamber and adhesion assays, respectively. U87 cells cultured in CD90^high^ CM exhibited a significantly greater migration ability than cells cultured in CD90^low^ CM and 0%DMEM (Fig. 3a). Furthermore, U87 cells were incubated with serum-free medium, CD90^low^ gbMSCs conditioned medium and CD90^high^ gbMSCs conditioned medium (0%DMEM, CD90^low^ CM, and CD90^high^ CM, respectively). We found that the proliferation ability of the U87 cells was significantly increased when incubated in CD90^high^ CM compared to that of the cells incubated in CD90^low^ CM and 0%DMEM (Fig. 3b). In addition, the adhesion assay revealed that the CD90^high^ CM significantly enhanced the adhesive capacity of the U87 cells compared to that of the CD90^low^ CM and 0%DMEM (Fig. 3c).

### CD90^low^ gbMSCs show strong angiogenesis and contribute to new tube formation in vitro

**Fig. 4:**
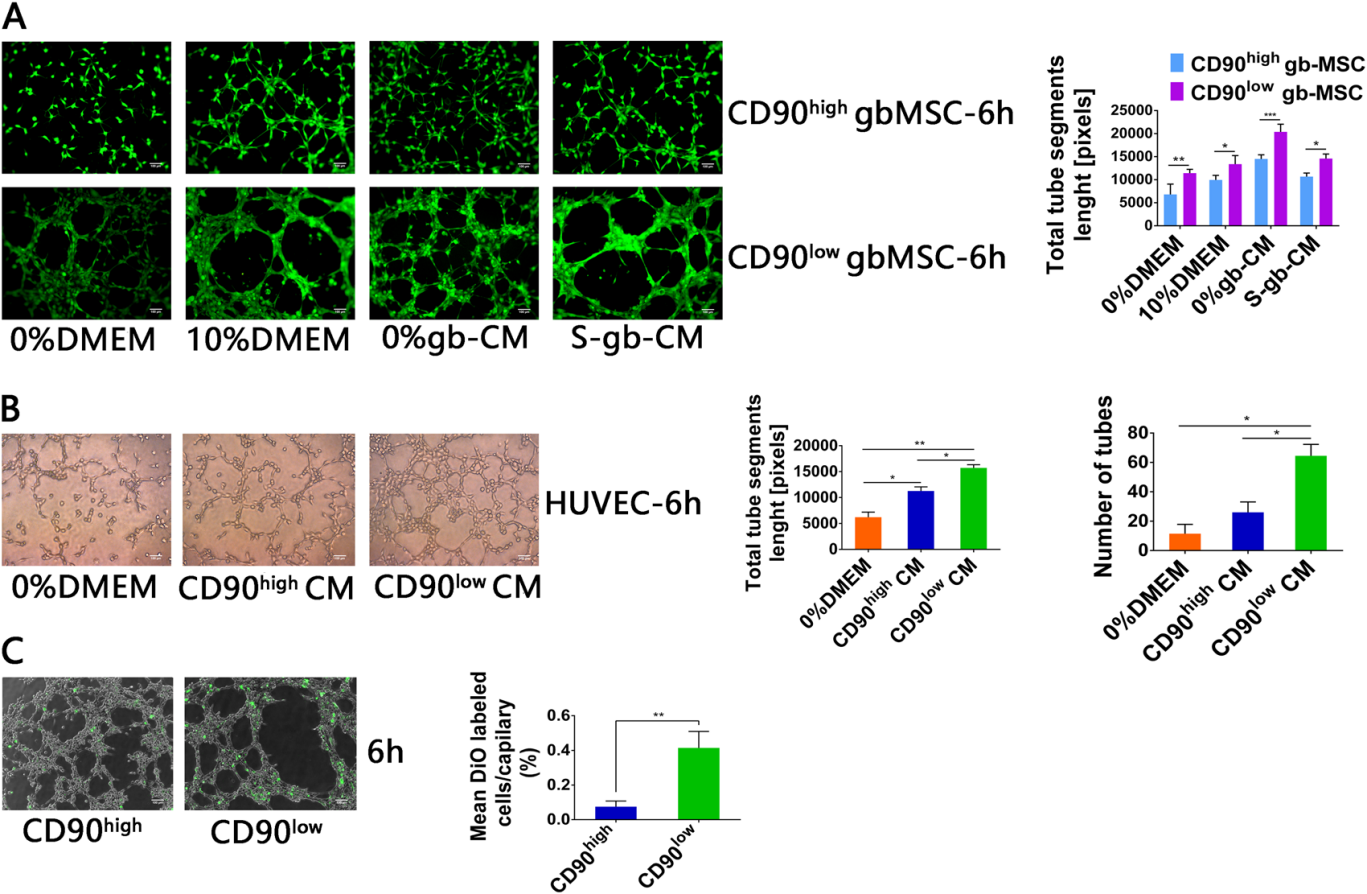
Tube formation capacity of gbMSCs and HUVECs incubated in different media. **a** Angiogenic capacity of CD90^high^ and CD90^low^ gbMSCs cultured in 0%DMEM, 10%DMEM, 0%gb-CM and S-gb-CM for 6 h on Matrigel (×100, scale bars = 100 µm). (*n* ≥ 3) *P < 0.05, **P < 0.01, ***P < 0.001. **b** Angiogenic capacity of HUVECs cultured in 0%DMEM, CD90^high^ CM and CD90^low^ CM for 6 h on Matrigel (×100, scale bars = 100 µm). (*n* ≥ 3) *P < 0.05, **P < 0.01. **c** Attachment capacity of DiO-labelled CD90^low^ and CD90^high^ gbMSCs onto vascular structures formed by HUVECs in 0%gb-CM (×100, scale bars = 100 µm). (*n* ≥ 3) *P < 0.05 and **P < 0.01

The involvement of MSC-derived human glioma in neovascularization is well established^6^. Thus, we focused on studies of the vascular formation ability of the gbMSC subtypes in glioblastoma-conditioned media. We found that tube formation at 6 h was significantly increased in the CD90^low^ gbMSCs incubated in different media (0%DMEM, 10%DMEM, 0%gb-CM, and S-gb-CM) compared to that of their CD90^high^ counterparts. The total tube segment lengths were also quantified; the tube segment lengths of the CD90^low^ gbMSCs were significantly longer than those of their CD90^high^ counterparts (Fig. 4a).

To study the capacity for angiogenesis generated by HUVECs cultured in the different media, HUVECs were seeded into wells and incubated with 0%DMEM, CD90^low^ CM and CD90^high^ CM. The tube networks were photographed at 6 h and analysed. The results showed the that angiogenic capacity of HUVECs cultured in CD90^low^ CM was significantly greater than that of the cells cultured in CD90^high^ CM and 0%DMEM (Fig. 4b).

Attachment of pericytes to ECs helps maintain and stabilize capillary-like structures. Therefore, we sought to elucidate the attachment capacity of CD90^low^ and CD90^high^ gbMSC-derived pericytes. Calcein AM-labelled CD90^low^ gbMSCs and HUVECs (1:2) and Calcein AM-labelled CD90^high^ gbMSCs and HUVECs (1:2) were co-seeded into the same medium and photographed at 6 h.The tube formation analyses showed that the attachment capacity of the CD90^low^ gbMSC-derived pericytes was better than that of CD90^high^ gbMSC-derived pericytes (Fig. 4c).

### Different functions of CD90^high^ and CD90^low^ gbMSCs in vivo

**Fig. 5:**
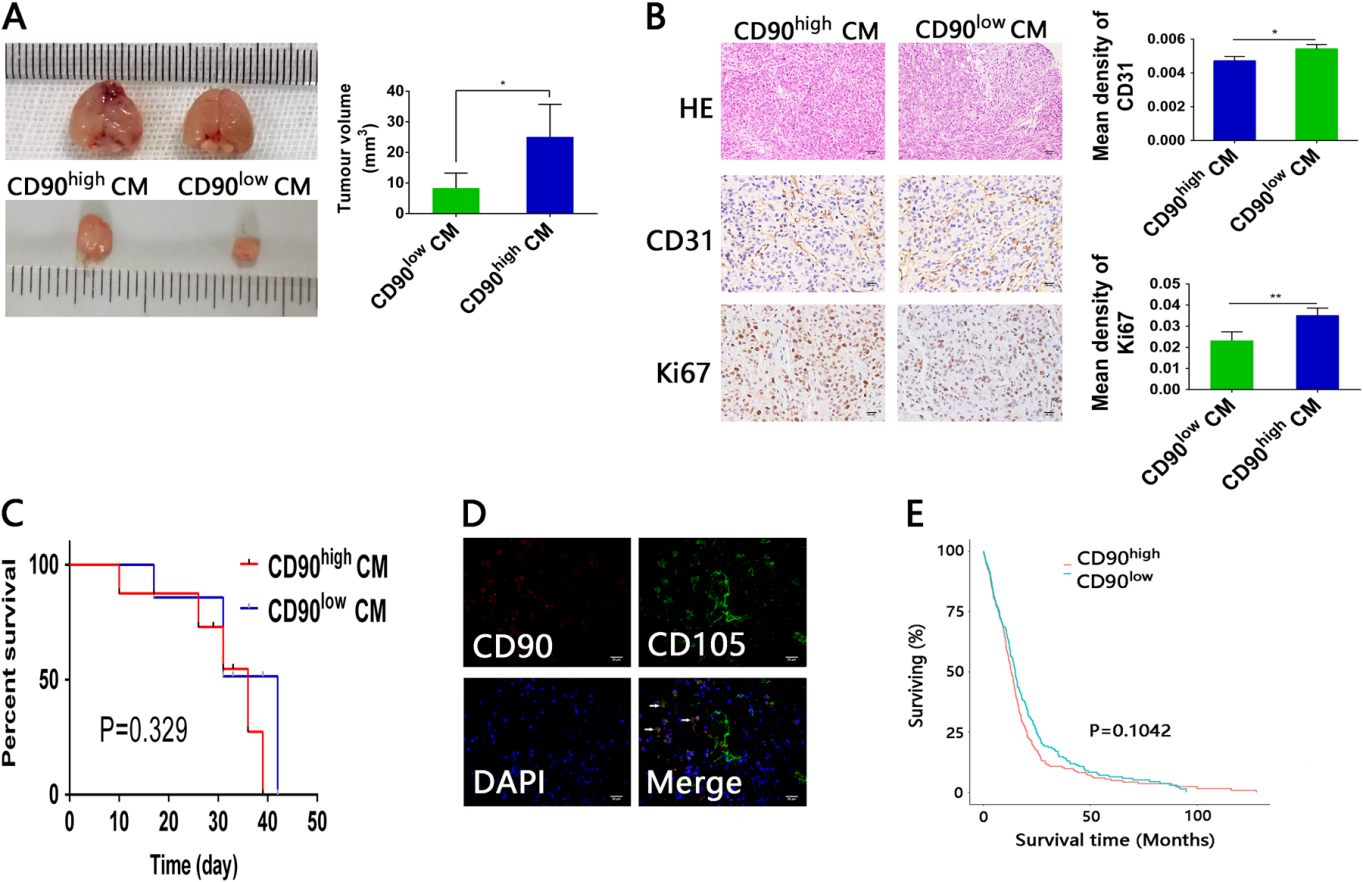
Conditioned media from CD90^high^ and CD90^low^ gbMSCs have different functions in vivo. **a** Representative mice from intracranial xenograft experiments in which U87 cells with CD90^high^ CM (left) or U87 cells with CD90^low^ CM (right) were injected into the right frontal lobes of nude mice. Obviously, the sizes of the CD90^high^ CM group tumours were greater than those of their CD90^low^ CM counterparts. *P < 0.05. **b** Both the CD90^high^ CM and CD90^low^ CM tumour sections were stained with HE (×200, scale bars = 50 μm). In the CD90^high^ CM and CD90^low^ CM tumour tissues, IHC was employed to detect CD31 and Ki-67 expression (×400, scale bars = 25 µm). (*n* ≥ 3) *P < 0.05, **P < 0.01. **c** Survival curves of glioma-bearing mice. The survival times of mice implanted with U87 cells cultured with CD90^high^ CM were not significantly shorter than those of mice implanted with U87 cells cultured in CD90^low^ CM. **d** Double staining for CD105 (green) and CD90 (red) revealed that CD105^+^CD90^−^ cells were located in the vessel walls, whereas CD105^+^CD90^+^ cells were located around the tumour parenchyma. (×400, scale bars = 25 µm). **e** Kaplan–Meier survival curves for patients with low and high CD90 expression. The survival of glioma patients with different CD90 expression levels in TCGA database was not significantly different

To demonstrate that CD90^high^ CM and CD90^low^ CM had different functions in vivo, U87 cells with CD90^high^ CM and CD90^low^ CM were implanted simultaneously into the brains of mice (*N* = 6 mice/group). The tumour size was significantly larger with CD90^high^ CM than with CD90^low^ CM (Fig. 5a), indicating that CD90^high^ CM increased the proliferation and/or tumourigenesis of U87 cells in vivo. CD31 expression in the CD90^low^ CM tumour specimens was significantly higher than that of their CD90^high^ CM counterparts, whereas Ki-67 expression was significantly higher in the CD90^high^ CM tumour specimens than CD90^low^ CM counterparts (Fig. 5b). However, the survival time of the mice implanted with U87 cells co-cultured with CD90^high^ CM was not significantly shorter than that of mice implanted with U87 cells cultured in CD90^low^ CM (Fig. 5c). Additionally, we found that CD90^**-**^CD105^**+**^ cells were mainly located in the vessel walls, whereas CD105^**+**^CD90^**+**^ gbMSCs were located around the tumour parenchyma specimens in human glioblastomas (Fig. 5d). Moreover, the overall survival of patients with gliomas from TCGA database was not significantly different when the patients were group based on their CD90 expression levels (Fig. 5e).

### Secretion of factors related to CD90^high^ and CD90^low^ gbMSCs

**Fig. 6:**
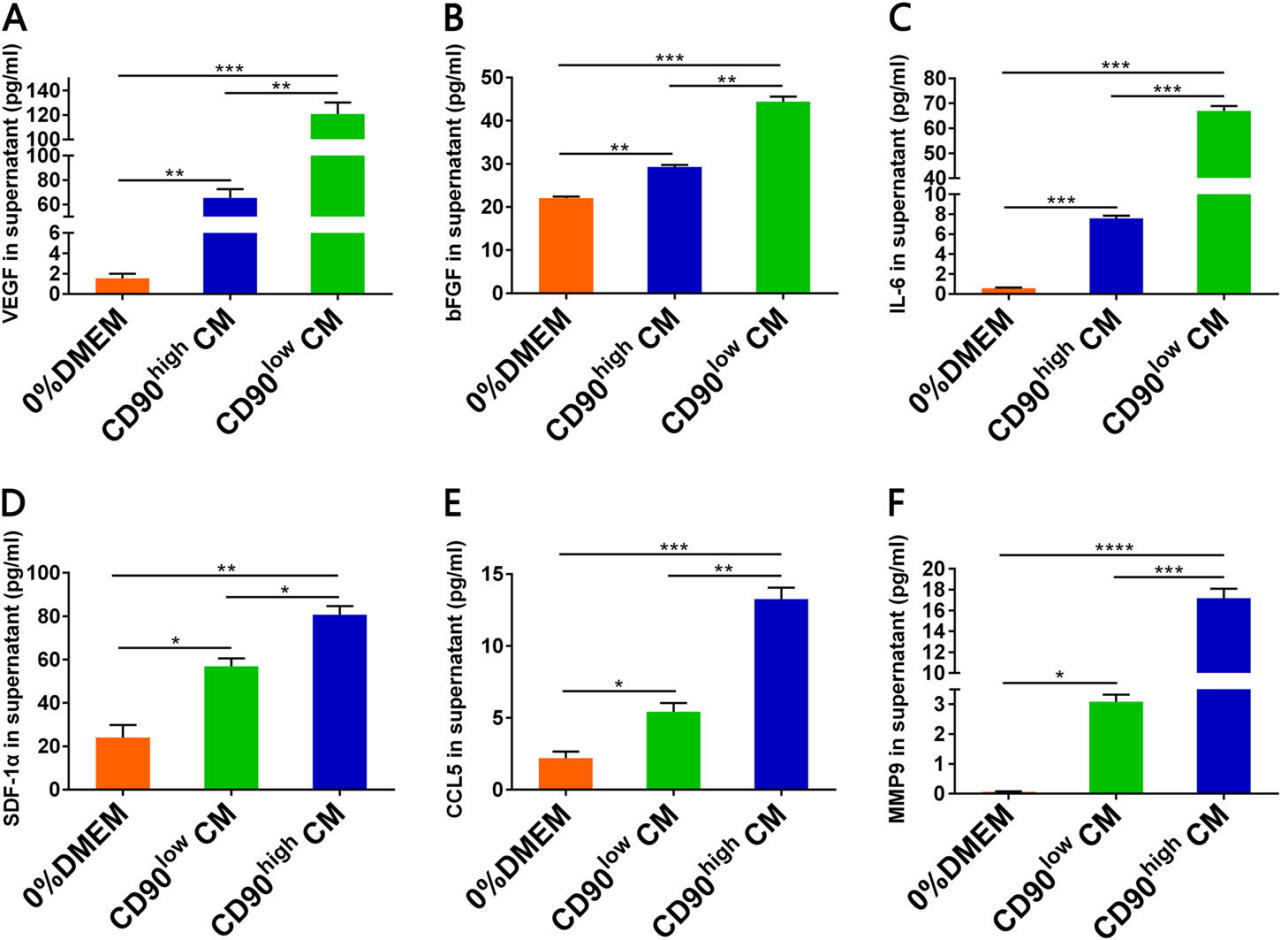
The VEGF, IL-6, bFGF, MMP9, CCL5 and SDF-1αlevels in different treatment media by ELISA. **a** The VEGF (*n* ≥ 3) levels were significantly higher in CD90^low^ CM compared to those in CD90^high^ CM and 0%DMEM. **b** The bFGF (*n* ≥ 3) levels were significantly higher in CD90^low^ CM compared to those in CD90^high^ CM and 0%DMEM. **c** The IL-6 (*n* ≥ 3) levels were significantly higher in CD90^low^ CM compared to those in CD90^high^ CM and 0%DMEM. * **d** The SDF-1α (*n* ≥ 3) levels were higher in CD90^high^ CM compared to those in CD90^low^ CM and 0%DMEM. **e** The CCL5 (*n* ≥ 3) levels were higher in CD90^high^ CM compared to those in CD90^low^ CM and 0%DMEM. **f** The MMP9 (*n* ≥ 3) levels were significantly higher in CD90^high^ CM compared to those in CD90^low^ CM and 0%DMEM. *P* < 0.05, **P < 0.01, ***P < 0.001 and ****P < 0.0001

To analyse the secretion of several factors in different media, supernatants were collected and evaluated by ELISA. We found that the VEGF, FGF-2, and IL-6 levels were higher in the CD90^low^ CM than in the CD90^high^ CM and 0%DMEM (Fig. 6a-c). Conversely, the SDF-1α, CCL5, and MMP9 levels were higher in the CD90^high^ CM than in the CD90^low^ CM and 0%DMEM (Fig. 6d-f).

### Differential lncRNA and mRNA expression in the CD90^high^ and CD90^low^ gbMSCs

**Fig. 7:**
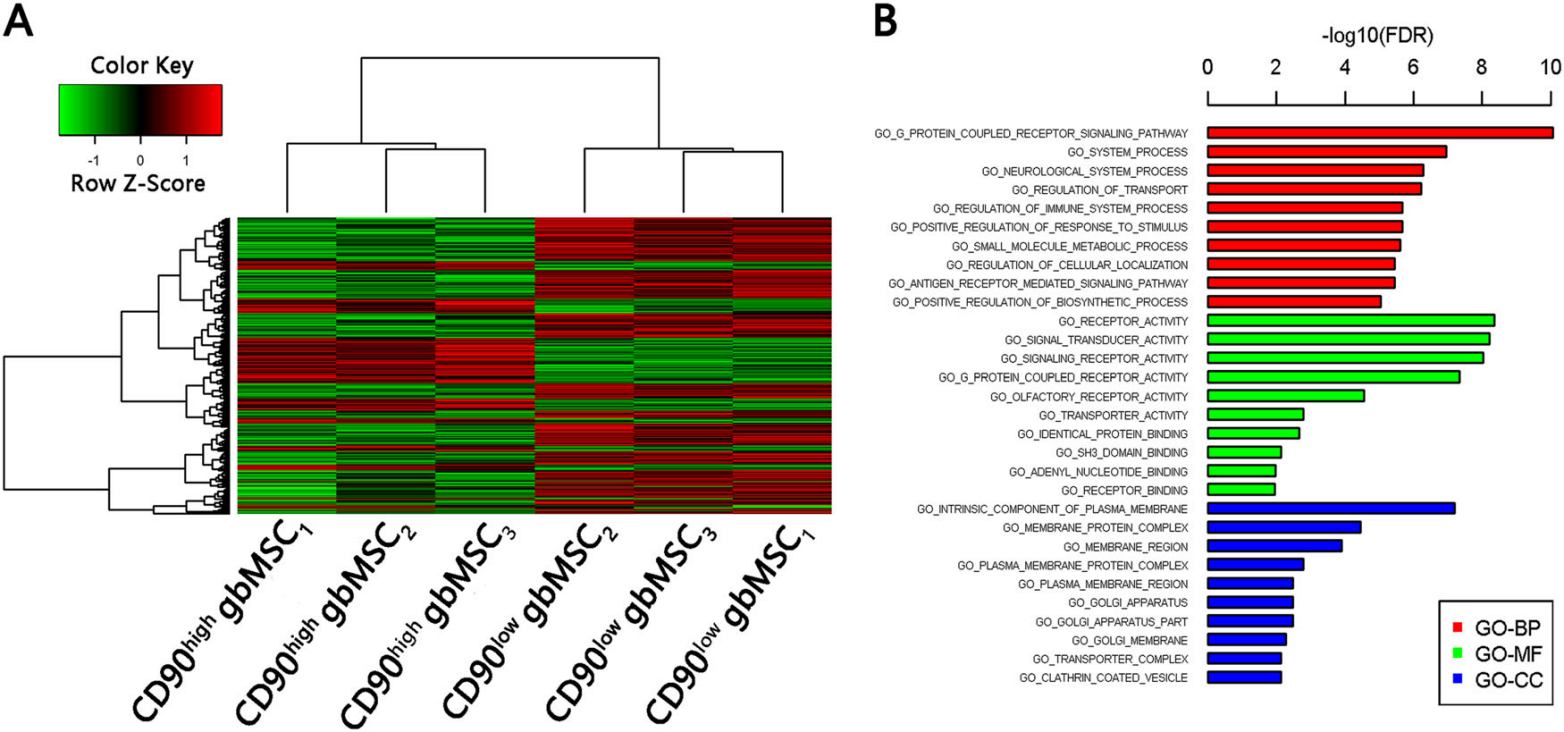
Clariom D expression profiles of CD90^high^ and CD90^low^ gbMSCs (*n* = 3). **a** Heatmap of differentially expressed lncRNAs from a microarray assay performed on CD90^high^ and CD90^low^ gbMSCs. ‘Red’ indicates high relative expression, and ‘green’ indicates low relative expression. **b** GO terms for the predicted targeted genes. *P* < 0.05 using the two-sided Fisher’s exact test was defined as statistically significant

Clariom D analysis of the CD90^high^ and CD90^low^ gbMSCs cultured in standard medium revealed different lncRNA and mRNA expression profiles. Subsequently, a test was performed to identify differentially expressed genes between the CD90^high^ and CD90^low^ gbMSCs, and a total of 4977 genes (2055 up regulated and 2922 down regulated in the CD90^high^ gbMSCs) were identified (Fig. 7a). Differentially expressed mRNA target genes were predicted using TargetScan, lncRNA.org and the LnRDBA database, and the results were applied to a gene ontology (GO) term analysis (Fig. 7b), including the biological process (BP), molecular function (MF) and cellular component (CC) categories. The predicted target genes were enriched in cell migration, proliferation, adhesion and angiogenesis.

## Discussion

In 1966, the existence of MSCs in bone marrow was first reported by Friendenstein et al.^11^. Thereafter, MSCs were widely and gradually researched in several fields, but studies were limited by the lack of a definitive marker for MSCs, which meant that the cell type could only be defined in terms of cellular morphology, surface markers and differentiation potential. In 2006, the International Society for Cellular Therapy (ISCT) defined the minimal criteria for MSCs according to their biological features^12^. Positive CD90 expression is one of the minimal criteria used to define MSCs, and MSCs expressing high CD90 levels can be found in tissues including bone marrow and adipose tissue^13^. In gliomas, tumour cells recruit MSCs from different tissues and corrupt them into gbMSCs to promote tumour progression^14^. Compared to their main BM-MSC resources, gbMSCs harbour genetic alterations, of which CD90 expression is one difference^6^. In the current study, we found two subpopulations (CD90^high^ gbMSCs and CD90^low^ gbMSCs) that could be sorted from all fresh glioma tissues (WHO II–IV gliomas). CD90^low^ gbMSCs were more abundant than CD90^high^ gbMSCs in the same glioma tissues. With the exception of CD90 expression, these two subpopulations showed similar cellular morphologies. The CD90^low^ gbMSCs had stronger proliferation and migration abilities than the CD90^high^ gbMSCs. Previous literature reported that angiogenic stimuli and mechanical stress led to a loss of CD90 expression in MSCs^15,16^. Our previous study reported that CD90^low^ gbMSCs could differentiate into pericytes and contribute to angiogenesis in the malignant glioma microenvironment^6^. Thus, tumour cells may influence CD90 expression on MSCs recruited to a glioma for angiogenesis, but tumour cells may recruit different gbMSC subpopulations from different resources for different hallmarks of malignant gliomas.

To date, MSCs from different sources have shown different abilities to promote or suppress the growth of glioma cells under different conditions^17–21^. In gliomas, tumour cells do not recruit MSCs into the tumour microenvironment as bystanders or inhibitorsof progression^5,14^. Previous studies found that high percentages of gbMSCs in tumour samples correlated with poor outcomes of patients with high-grade gliomas. Additionally, gbMSCs could increase the tumourigenicity and maintain the stemness of glioma stem cells^9^. In the current study, we investigated the different roles of two subpopulations (CD90^high^ and CD90^low^ gbMSCs) in aggressive progression of gliomas. Conditioned medium from CD90^high^ gbMSCs induced faster proliferation and stronger migration and adherence of U87 cells in vitro, and injection of conditioned medium from CD90^high^ gbMSCs resulted in a larger tumour volume and more Ki-67-labelled tumour cells in an animal model. Conversely, the CD90^low^ gbMSCs had a strong ability to differentiate into pericytes and induce tube formation in vitro, and conditioned medium from the CD90^low^ gbMSCs induced more capillary-like endothelial cell structures. In animal models, injection of conditioned medium from CD90^low^ gbMSCs induced more CD31-expressing vessels. In human glioblastoma tissues, CD105^**+**^CD90^−^ cells were located in the vessel walls, and CD105^**+**^CD90^**+**^ cells were located around the tumour parenchyma. These data suggested that CD90^high^ gbMSCs mainly promoted tumour cell proliferation, where as CD90^low^ gbMSCs mainly differentiated into pericytes and contributed to angiogenesis. However, gbMSCs are not the sole players in the tumourigenicity of malignant gliomas, and the overall survival of patients with gliomas in TCGA database and tumour-bearing mice is not significantly different between groups with different CD90 expression levels.

Cytokines are a very important component of the cross-talk between tumour cells and the tumour microenvironment^3,6^. Previous studies showed that cytokines and secreted exosomes were important for the effect of gbMSCs on the tumourigenicity of malignant gliomas^9,22^. Therefore, cytokines and related lnRNAs were compared between CD90^high^ and CD90^low^ gbMSCs in the current study. CD90^low^ gbMSCs produced higher levels of the angiogenesis factors VEGF, bFGF and IL-6, and CD90^high^ gbMSCs produced higher levels of the growth factors SDF-1α, CCL5, and MMP9. These results are consistent with previous findings^23–28^. To explore their gene profiles, we analyse differences in expression based on the Clariom D assay and the predicted gene ontologies of the CD90^high^ and CD90^low^ gbMSCs. We identified specific lncRNA, mRNA and target gene pairs in the CD90^high^ and CD90^low^ gbMSCs. Previous reports indicated important links between the G protein-coupled receptor pathway and tumour proliferation^29–32^ and angiogenesis^33–35^. In future studies, we will identify the specific lncRNA, mRNA and target gene pairs that are significantly different between the CD90^low^ and CD90^high^ gbMSCs and promote tumour proliferation and angiogenesis.

In conclusion, we elaborately sorted two subpopulations (CD90^high^ and CD90^low^ gbMSCs) from gbMSCs. Both subpopulations had cellular morphologies and surface markers similar to those of classical MSCs but showed slightly different biological features and played enormously different roles in the glioma microenvironment. Both in vivo and in vitro, CD90^high^ gbMSCs significantly promoted glioma cell growth, and the CD90^low^ gbMSCs promoted angiogenesis via pericyte transition. Additionally, we provided evidence that the expression patterns of secreted factors and related lncRNAs in these two subpopulations might affect their roles in glioma progression. However, determining the underlying mechanism requires further exploration in more detail. Therefore, these results revealed that future therapies targeting the two distinct gbMSC subpopulations should use different strategies.
