# Correction: CD90 determined two subpopulations of glioma-associated mesenchymal stem cells with different roles in tumour progression

**DOI:** 10.1038/s41419-026-08786-y

**Published:** 2026-05-18

**Authors:** Qing Zhang, Dong-Ye Yi, Bing-Zhou Xue, Wan-Wan Wen, Yin-Ping Lu, Ahmed Abdelmaksou, Min-xuan Sun, De-tian Yuan, Hong-Yang Zhao, Nan-Xiang Xiong, Wei Xiang, Peng Fu

**Affiliations:** 1 Department of Neurosurgery,Union Hospital, Tongji Medical College, Huazhong University of Science and Technology, Wuhan, 430022 China; 2 Department of Cardiology, Beijing Anzhen Hospital, Capital Medical University, No. 2, Anzhen Road, Chaoyang District, Beijing, 100029 China; 3 Institute of Infection and Immunology, Union Hospital, Tongji Medical College, Huazhong University of Science and Technology, Wuhan, 430022 China; 4 Department of Neurosurgery, Faculty of Medicine, Helwan University, Cairo, 11435 Egypt; 5 Jiangsu Key Lab of Medical Optics, Suzhou Institute of Biomedical Engineering and Technology, Chinese Academy of Sciences, Suzhou, 215163 China

Correction to: *Cell Death & Disease* 10.1038/s41419-018-1140-6, published online 27 October 2018

Since the publication of this paper, the authors have noted that Figure 4A is incorrect. It was mistakenly exchanged in the final article. This has now been rectified and the corrected article appears online together with this corrigendum. The authors would like to apologize for any inconvenience this may have caused.

**Fig. 1:**
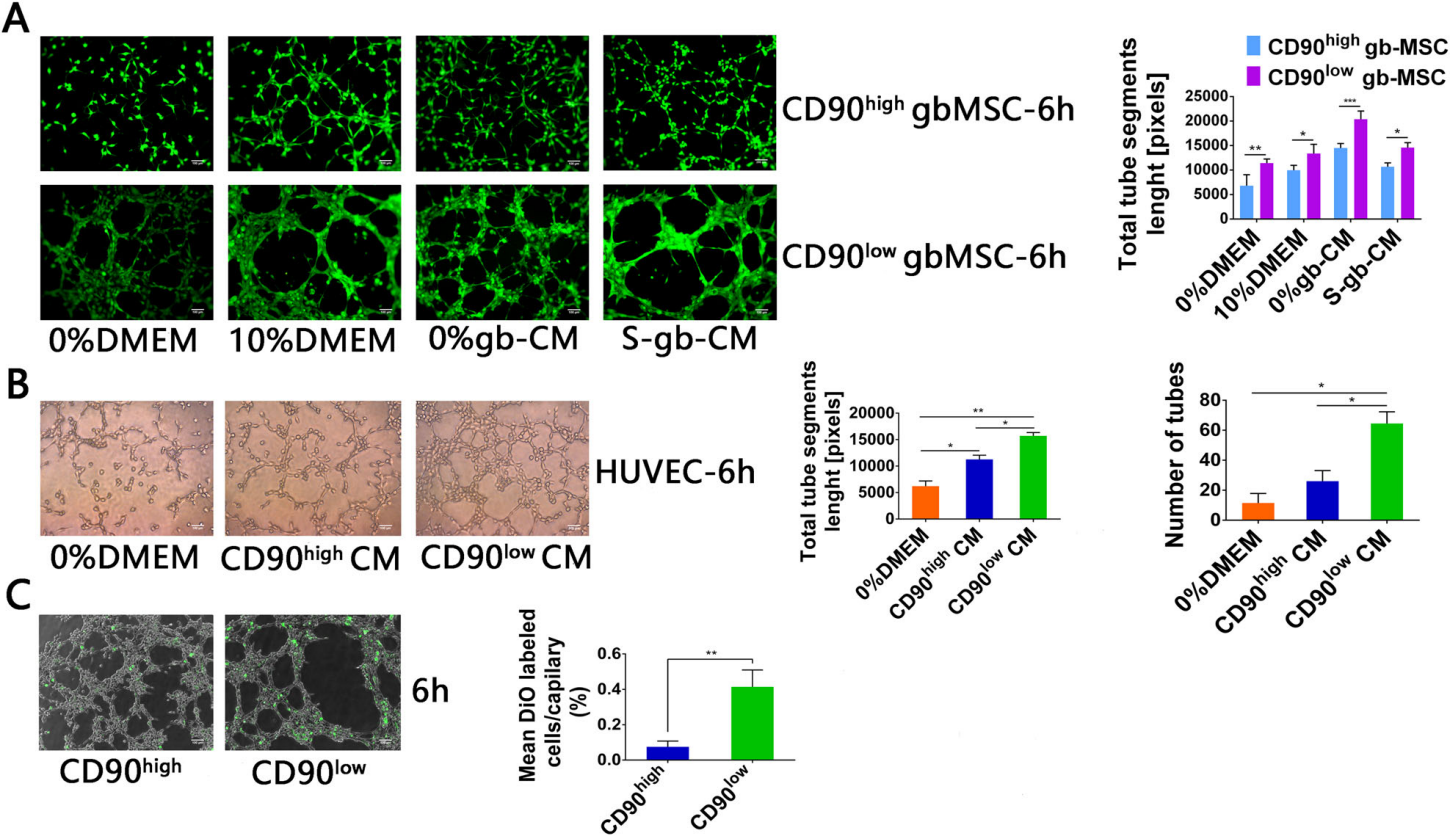
Tube formation capacity of gbMSCs and HUVECs incubated in different media. **A**. Angiogenic capacity of CD90^high^ and CD90^low^ gbMSCs cultured in 0%DMEM, 10%DMEM, 0%gb-CM and S-gb-CM for 6 h on Matrigel (×100, scale bars = 100 µm). (*n* ≥ 3) *P < 0.05, **P < 0.01, ***P < 0.001. **B**. Angiogenic capacity of HUVECs cultured in 0%DMEM, CD90^high^ CM and CD90^low^ CM for 6 h on Matrigel (×100, scale bars = 100 µm). (*n* ≥ 3) *P < 0.05, **P < 0.01. **C**. Attachment capacity of DiO-labelled CD90^low^ and CD90^high^ gbMSCs onto vascular structures formed by HUVECs in 0%gb-CM (×100, scale bars = 100 µm). (n ≥ 3) *P < 0.05 and **P < 0.01.

